# *Moringa oleifera* Lam.: A Nutritional Powerhouse with Multifaceted Pharmacological and Functional Applications

**DOI:** 10.3390/life15060881

**Published:** 2025-05-29

**Authors:** Natalina Panova, Anelia Gerasimova, Galia Gentscheva, Stoyanka Nikolova, Lubomir Makedonski, Margarita Velikova, Abdessamad Beraich, Abdelmonaem Talhaoui, Nadezhda Petkova, Daniela Batovska, Krastena Nikolova

**Affiliations:** 1 Department of Physics and Biophysics, Faculty of Pharmacy, Medical University of Varna, 84 Tzar Osvoboditel, 9000 Varna, Bulgaria; panova@mu-varna.bg; 2 Department of Chemistry, Faculty of Pharmacy, Medical University of Varna, 84 Tzar Osvoboditel, 9000 Varna, Bulgaria; anelia.gerasimova@mu-varna.bg (A.G.); makedonski@mu-varna.bg (L.M.); 3 Department of Chemistry and Biochemistry, Medical University-Pleven, 1 Saint Kliment Ohridski Street, 5800 Pleven, Bulgaria; 4 Department of Organic Chemistry, Faculty of Chemistry, University of Plovdiv, 4000 Plovdiv, Bulgaria; tanya@uni-plovdiv.bg; 5 Department of Physiology and Pathofysiology, Faculty of Medicine, Medical University of Varna, 55 Marin Drinov, 9000 Varna, Bulgaria; margarita.stefanova@mu-varna.bg; 6 Laboratory of Environment and Applied Chemistry (LCAE), Team: Physical Chemistry of the Natural Resources and Processes, Department of Chemistry, Faculty of Sciences, Mohamed First University, Oujda 60000, Morocco; abdessamadberaich721@gmail.com (A.B.); a.talhaoui@ump.ac.ma (A.T.); 7 Department of Organic Chemistry and Inorganic Chemistry, University of Food Technologies, 26 Maritsa Blvd., 4002 Plovdiv, Bulgaria; nadezhda_petkova@uft-plovdiv.bg; 8 Institute of Chemical Engineering, Bulgarian Academy of Sciences, Acad. G. Bonchev Str., Bl. 103, 1113 Sofia, Bulgaria; danielabatovska@gmail.com

**Keywords:** *Moringa oleifera*, nutrients, phytochemistry, antioxidant activity, pharmacological properties, traditional medicine, functional foods, cosmeceuticals

## Abstract

*Moringa oleifera*, often referred to as the “miracle tree”, has gained widespread recognition for its exceptional nutritional profile and broad pharmacological potential. This review provides a comprehensive synthesis of the plant’s botanical characteristics, taxonomy, cultivation practices, and biochemical composition. Special emphasis is placed on its rich content of bioactive secondary metabolites-such as flavonoids, alkaloids, phenolic acids, saponins, isothiocyanates, and glucosinolates-which underlie its diverse therapeutic effects. The paper compiles and analyzes evidence from over 200 peer-reviewed studies, documenting antioxidant, anti-inflammatory, antimicrobial, antidiabetic, anticancer, hepatoprotective, neuroprotective, and anti-obesity effects, among others. For instance, leaf extracts have demonstrated potent antioxidant and antidiabetic effects in both animal models and clinical trials, while seed-derived isothiocyanates have shown significant antibacterial and anticancer activity. In addition, clinical and in vivo data support *M. oleifera*’s role in fertility regulation, cardiovascular protection, and neurodegenerative disease mitigation. Beyond its medicinal applications, the review highlights its growing use in functional foods, dietary supplements, and cosmeceutical products, reflecting its commercial and industrial relevance. By consolidating findings across disciplines, this review underscores the multifaceted value of *M. oleifera* as a nutraceutical and therapeutic resource.

## 1. Introduction

*Moringa oleifera* Lam, commonly known as the “miracle tree”, is a fast-growing, drought-tolerant species native to the Indian subcontinent and now cultivated extensively across tropical and subtropical regions [1]. Its reputation as a multipurpose plant stems from the fact that nearly every part—including leaves, seeds, pods, bark, flowers, and roots—has traditional or modern applications in food, medicine, agriculture, and cosmetics. The plant thrives in marginal soils, requires minimal inputs, and tolerates prolonged dry periods, making it an important candidate for sustainable agriculture in regions facing malnutrition, climate variability, and land degradation [2].

In recent decades, *M. oleifera* has drawn increasing global attention due to its exceptional nutritional and pharmacological potential. Given the accelerating volume of research on *M. oleifera*, driven by its broad utility and increasing global relevance, there is a pressing need to critically evaluate and synthesize emerging findings. Such an integrative review will help contextualize recent advances, highlight knowledge gaps, and facilitate targeted investigations across scientific, clinical, and agricultural domains. The leaves, in particular, are valued for their high content of proteins, essential amino acids, vitamins (A, B-complex, C, and E), and minerals such as calcium, potassium, iron, and magnesium [2]. These properties have led to its incorporation into feeding programs, food fortification strategies, and dietary supplements, particularly in low-resource settings.

Phytochemical analyses of *M. oleifera* have identified a broad range of bioactive compounds, including flavonoids, phenolic acids, glucosinolates, carbamates, tannins, and saponins [3,4,5]. These constituents contribute to the plant’s wide spectrum of pharmacological activities, such as antioxidant, anti-inflammatory, antimicrobial, antidiabetic, anticancer, hepatoprotective, cardioprotective, and neuroprotective effects [2,3,4,5,6]. Extracts from various plant parts have shown promising results in the prevention and management of chronic diseases, including diabetes, cancer, and neurodegenerative disorders [7]. Additional studies have reported antiepileptic, wound-healing, and immunomodulatory properties [6,8]. Moreover, the high oleic acid content of the leaves supports their use in cosmeceutical formulations, providing moisturizing, emollient, and skin-protective effects [9].

As a result of these combined attributes, *M. oleifera* has become a focal point in the development of functional foods, herbal therapeutics, and natural personal care products. Its inclusion in climate-resilient cropping systems and its use in reforestation, soil rehabilitation, and carbon sequestration initiatives further enhance its significance on both the health and environmental fronts.

Despite the growing number of reviews on *M. oleifera* L., most have concentrated on specific, narrowly defined themes. For example, Berg and Kuipers systematically assessed its antibacterial properties [10], while Chiș et al. focused on the anti-inflammatory mechanisms of key bioactive compounds such as quercetin, kaempferol, and isothiocyanates [11]. Other authors reviewed its nutraceutical and cosmeceutical applications [12], Abdelwanis et al. addressed its industrial and agricultural value [13], particularly in Egypt [14,15], and Elsadek et al. explored its regenerative and osteogenic effects in oral medicine [14]. Singh et al. examined its anticancer potential from a phytochemical and pharmacological perspective [15], while Anzano et al. provided a broader overview of its botany and medicinal applications [16]. However, an integrated and updated synthesis that bridges these domains is still lacking.

This review addresses that gap by offering a comprehensive, multidisciplinary analysis of *M. oleifera*, covering its botanical identity, taxonomy, cultivation strategies, phytochemical diversity, and biological activities, with particular emphasis on its role as a functional food crop and its agronomic adaptability beyond tropical regions. By consolidating recent findings from diverse research fields, we aim to support researchers, agricultural practitioners, and product developers in harnessing the full nutritional, therapeutic, and ecological potential of *M. oleifera* in both developing and developed contexts.

## 2. Methodology

This review is based on a comprehensive literature search conducted to collect and analyze scientific publications related to *M. oleifera* from 2010 up to February 2025. Databases including PubMed, Scopus, Science Direct, Web of Science, and Google Scholar were used to identify relevant studies. The search terms included combinations of keywords such as “*Moringa oleifera*”, “nutritional composition”, “phytochemistry”, “pharmacological activity”, “bioactive compounds”, “antioxidant”, “anti-inflammatory”, “traditional medicine”, and “functional foods”.

Only peer-reviewed journal articles published in English were included. Review articles and original research papers describing the botanical, nutritional, chemical, pharmacological, or commercial aspects of *M. oleifera* were considered. Studies based on in vitro, in vivo, or clinical evaluations were prioritized when discussing pharmacological effects. Articles were excluded if they were duplicate entries, non-peer-reviewed materials, or outside the scope of the review.

The selected studies were critically evaluated and organized thematically to provide a structured synthesis of the current knowledge on *M. oleifera*, with attention to both traditional uses and modern applications.

## 3. Botanical Identity, Taxonomy, and Cultivation

### 3.1. Botanical Description

*M. oleifera* is a fast-growing, deciduous tree that typically reaches up to 8 m in height. It has a twisted trunk with smooth, dark gray to yellow bark and an umbrella-shaped crown. The compound leaves are bipinnate or tripinnate, with ellipsoid leaflets that are dark green on the upper surface and pale green underneath, measuring up to 2.5 cm in length. The plant produces fragrant, creamy-white flowers arranged in loose inflorescences up to 15 cm long. Its fruit is a long, three-sided capsule commonly referred to as a “drumstick”, measuring up to 90 mm in length and 12 mm in width, containing light brown seeds that germinate within a week under suitable conditions [17].

Common names for *M. oleifera* vary widely across countries and languages, reflecting its broad geographic distribution and cultural importance [18,19]. These are presented in Table 1.

### 3.2. Taxonomy

The taxonomic classification of *M. oleifera* is summarized in Table 2, [20,21,22].

The species of the genus *Moringa* include: *M. oleifera*, *M. concanensis*, *M. drouhardii*, *M. arborea*, *M. borziana*, *M. hildebrandtii*, *M. longituba*, *M. pygmaea*, *M. rivae*, *M. ruspoliana*, *M. ovalifolia*, *M. peregrina*, and *M. stenopetala.*

### 3.3. Cultivation and Agronomical Practices

#### 3.3.1. Growing Conditions

*M. oleifera* is a highly adaptable species that thrives in tropical and subtropical regions, growing well from sea level up to 1000 m in elevation. It tolerates a wide range of temperatures—from 12 to 40 °C—and can withstand short-term extremes as low as 1–3 °C or as high as 48 °C [23]. It grows in diverse soils—including sandy, loamy, and semi-arid types—but performs best in well-drained soils with near-neutral pH, tolerating pH values between 5.0 and 9.0. It is also capable of surviving in nutrient-poor and degraded soils, making it suitable for cultivation in areas impacted by land degradation or climate stress [24].

#### 3.3.2. Cultivation

Propagation of *M. oleifera* can be achieved through seeds, hardwood cuttings, or nursery-grown transplants. Seeds germinate within two weeks when sown at shallow depths (up to 2 cm), whereas hardwood cuttings (1–2 m long, 4–16 cm thick) taken from mature trees during the rainy season root easily under moist conditions [24]. Trees grown from seeds develop deeper root systems, which enhance drought resilience, while cuttings establish more rapidly but produce shallower roots [23,25]. Germination and vegetative growth are optimal at day/night temperatures of approximately 30/20 °C [26].

Due to its rapid growth—up to 3 m in just three months—*M. oleifera* supports multiple harvest cycles annually. Pruning or pollarding is commonly practiced promoting lateral branching, facilitating harvest, and improving regrowth. Moderate pruning, in particular, has been shown to significantly increase leaf biomass compared to light or heavy pruning [26].

Planting density is tailored to the production goal:

- Planting density is usually tailored to the production objective. For example, for intensive leaf production: spacing is 10–20 cm, with harvest every 35–45 days, and requires irrigation and fertilization; for semi-intensive systems: spacing is approximately 50 × 100 cm, with harvest every 50–60 days and moderate inputs; for agroforestry systems: 2–4 m between rows, designed for low-input integration into wider farming systems.
- Yields vary widely depending on genotype, climate, and spacing, with intensive plantations producing between 40 and 580 metric tons of fresh biomass per hectare per year [23]. Shoots are typically harvested at 0.5–1 m height to stimulate regrowth, while harvesting individual leaves—although faster—may reduce vigor over time.

For seed production, wider spacing (2.5–3 m) is recommended to maximize pod development. Pods mature about three months after flowering and must be harvested promptly to avoid seed loss. A single tree can yield 15,000–25,000 seeds per year, depending on cultivar and environmental conditions [27,28].

Beyond traditional tropical zones, *M. oleifera* has demonstrated excellent adaptability to temperate climates. In Bulgaria, Indian-developed PKM1 and PKM2 cultivars have been successfully introduced under monitored greenhouse and field conditions, allowing leaf harvests at a plant height of ~50 cm [24,29,30,31,32]. Similarly, trials in Portugal and Spain have shown the species can be cultivated in Mediterranean climates, provided frost protection is in place during colder months [33,34]. In Portugal, *M. oleifera* has been successfully cultivated under temperate conditions, demonstrating good agronomic performance in Mediterranean climates. Its high carbon dioxide sequestration capacity-reported to be up to 20 times greater than that of typical vegetation—further supports its potential as a sustainable crop in climate-smart agriculture [34].

## 4. Nutritional Profile

*M. oleifera* leaves are widely acknowledged for their exceptional nutritional richness, containing over 90 phytonutrients, including essential amino acids, proteins, vitamins, and minerals. Among these, vitamins have been extensively studied across different leaf forms—fresh, dried, and powdered. Leaf powder, commonly available in health food stores, is typically derived from shade- or sun-dried leaves [35].

Fresh *M. oleifera* leaves are particularly rich in vitamins E, A, and C. Vitamin E is the most abundant, with concentrations reaching 448 mg/100 g in raw leaves—significantly higher than any other vitamin. Although some loss occurs during drying, powdered leaves still retain a substantial amount (113 mg/100 g). In comparison, fresh leaves contain approximately four times more vitamin A than carrots and seven times more vitamin C than oranges [35]. In addition, *M. oleifera* leaves provide notable amounts of B vitamins, including pyridoxine (B6), nicotinic acid (B3), and folic acid (B9), which support various metabolic and cellular functions [36]. Table 3 summarizes the concentrations of key vitamins across raw, dried, and powdered leaf forms [37].

**Figure 1:**
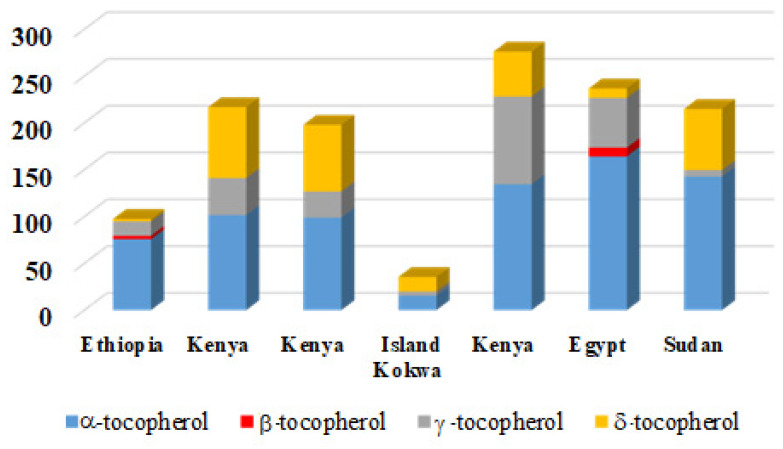
The content of individual vitamin E isomers in *M. oleifera* oil mg/kg.

The vitamin content of *M. oleifera* also shows considerable geographical variation. For instance, vitamin C levels range from 0.04% to 0.12%, with the highest values reported in samples from the United Kingdom, Jordan, and South Africa, and undetectable levels in samples from Nigeria [38]. A similar trend is observed for tocopherol content in *M. oleifera* oil: the highest concentrations of α- and β-tocopherol have been reported in samples from Ethiopia and Kenya, while γ-tocopherol levels show considerable variability, ranging from 4.47 to 93.70 mg/kg [39,40,41,42,43,44]. For example, Pluhackova et al. reported 2.6 mg/kg of β-tocopherol in Ethiopian samples [39], whereas Lalas et al. found a significantly higher level of 15.5 mg/kg in Kenyan samples [41]. Such variation is likely attributable to differences in plant genotype, environmental conditions, and harvest timing (Figure 1).

**Figure 2:**
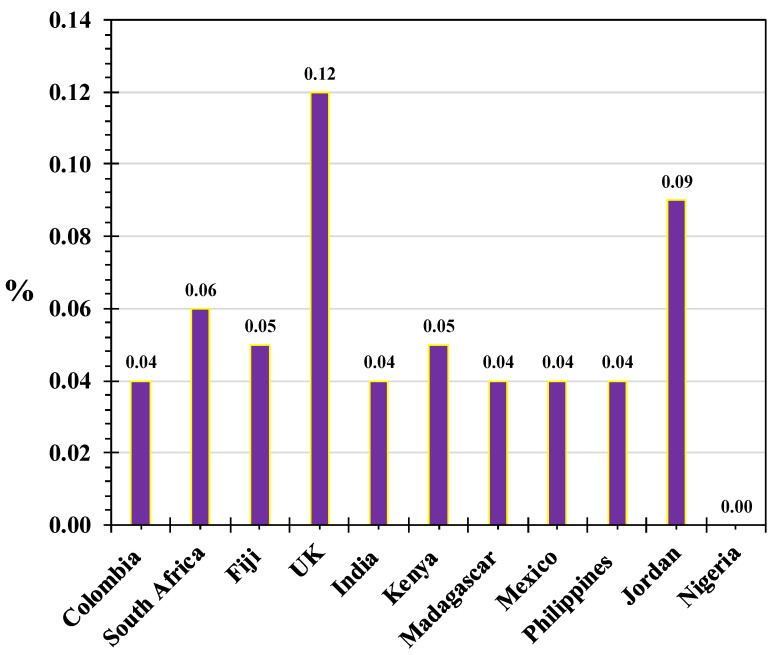
Relative percentage of the presence of the vitamin C identified in the different *M. oleifera* samples from different.

The vitamin C content of *M. oleifera* leaves has been studied in different countries [38]. The data are presented in Figure 2. The relative content varies in the range of 0.04 to 0.12%. The highest content is in the United Kingdom, followed by Jordan and South Africa. There is no vitamin C in samples from Nigeria.

**Figure 3:**
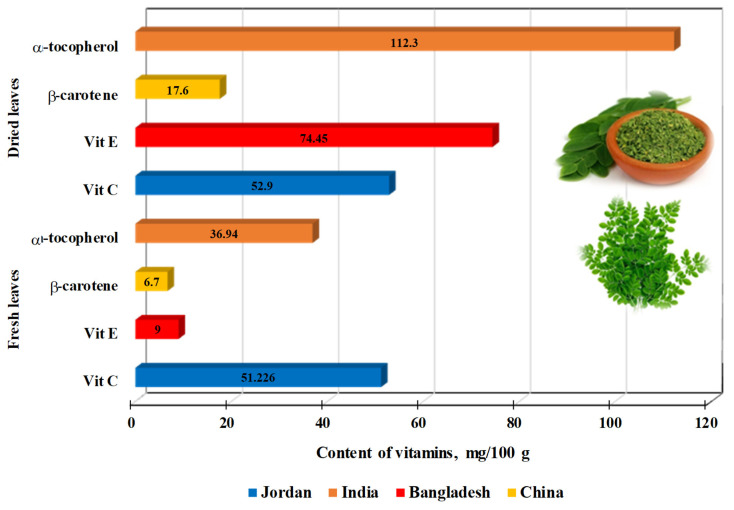
The content of vitamins in fresh and dried leaves of *M. oleifera* plant mg/100 g.

Figure 2 summarizes the results for vitamins and tocopherols in fresh and dried *M. oleifera* leaves [45]: The vitamin C content in both fresh and dried leaves is nearly identical. However, in the case of vitamin E, the concentrations of α- and β-tocopherols are 8.3, 3, and 2.6 times higher in dried leaves compared to fresh ones (Figure 3).

*M. oleifera* leaves are also an excellent source of essential minerals (Table 4), particularly calcium and potassium, both critical for muscle contraction and nerve signaling. High calcium levels support bone health, while magnesium plays a key role in energy metabolism. The leaves also contain notable amounts of iron, essential for oxygen transport and red blood cell formation. According to Gopalan et al. [46], *M. oleifera* leaves provide three times more potassium than bananas, four times more calcium than milk, and twice the protein content of yogurt. Dried leaves are particularly rich in iron, 25 times more than spinach [36].

A comparative review by Kashyap et al. [37] further confirmed that dried leaves contain the highest concentrations of phosphorus, magnesium, iron, and potassium, along with significantly greater levels of fats, fiber, carbohydrates, and caloric content than fresh leaves and extract powders. This enhanced nutrient density underscores the value of dried *M. oleifera* leaves as a concentrated dietary supplement.

**Figure 4:**
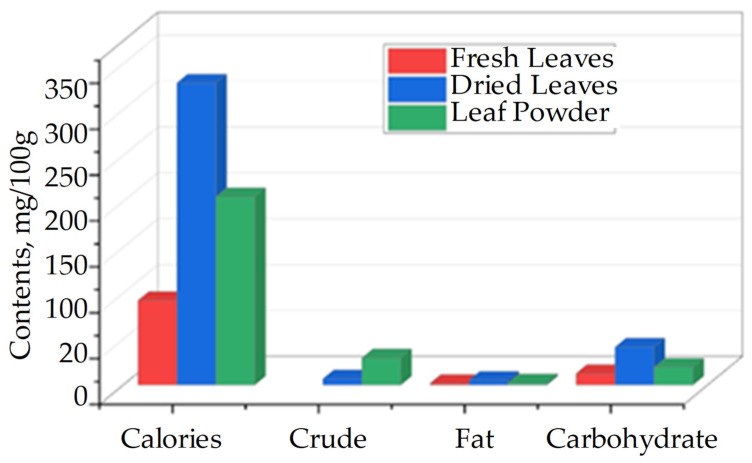
Nutrient content in fresh, dried leaves, and extract of *M. oleifera* [37].

The protein content of dried *M. oleifera* leaves reaches approximately 29.4 g/100 g, making them a superior plant-based protein source, exceeding even chia seeds in protein density [30]. Their caloric value ranges from 205 to 350 kcal per 100 g, depending on processing and origin (Figure 4).

Amino acid profiling has revealed that different plant parts of *M. oleifera* exhibit distinct profiles: leucine is predominant in both the stem (5.12 g/100 g) and the root (5.33 g/100 g), while arginine is most abundant in the leaves (8.22 g/100 g) [31], underscoring the plant’s nutritional diversity.

**Figure 5:**
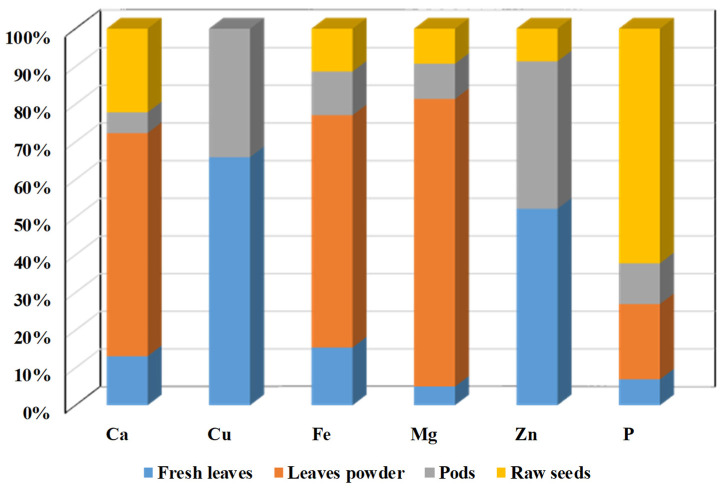
Percentage ratio of mineral content in different morphological organs of the *Moringa oleifera* [48].

The data presented in Figure 5 indicate that the ratio of mineral content differs among fresh leaves, raw seeds, powdered leaves, and pods. In fresh leaves, copper and zinc are predominant, whereas in powdered (dried) leaves, calcium, iron, and magnesium are present in the highest concentrations. Raw seeds are particularly rich in phosphorus.

## 5. Phytochemical Composition and Associated Bioactivities

The phytochemical composition of *M. oleifera* varies significantly across different plant parts and extraction methods (Table 5). Aqueous and alcoholic extracts of leaves, seeds, flowers, roots, and pods have yielded a wide spectrum of secondary metabolites with established or potential bioactivity. The major classes of compounds include flavonoids, carbamates, glucosinolates, and phenolic acids, each exhibiting organ-specific profiles and contributing to distinct therapeutic effects [3,49,50].

**Figure 6:**
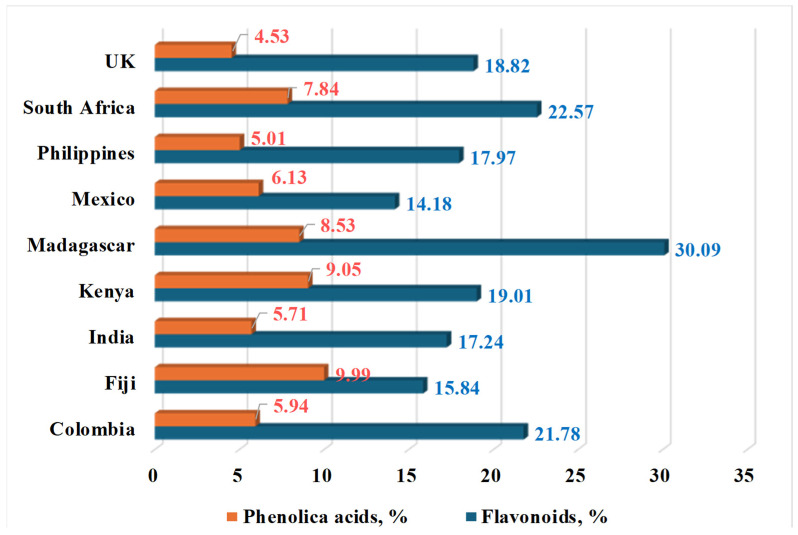
Percentage content of flavonoids and phenolic acids in *M. oleifera* leaf extract from different countries.

Figure 6 compares the total content of phenolic acids and flavonoids in plants from different countries. The data is from research by [38].

**Figure 7:**
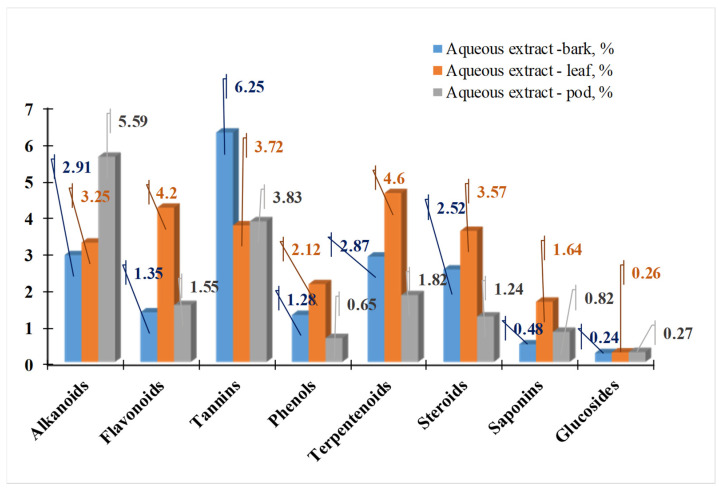
Compounds in Water extracts of bark, leaves, and pods of *M. oleifera.*

**Figure 8:**
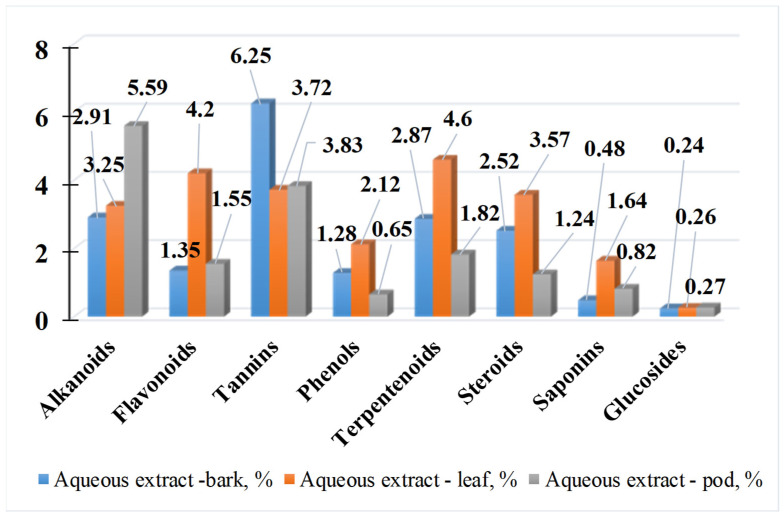
Compounds in Ethanol extracts of bark, leaves, and pods of *M. oleifera*.

**Figure 9:**
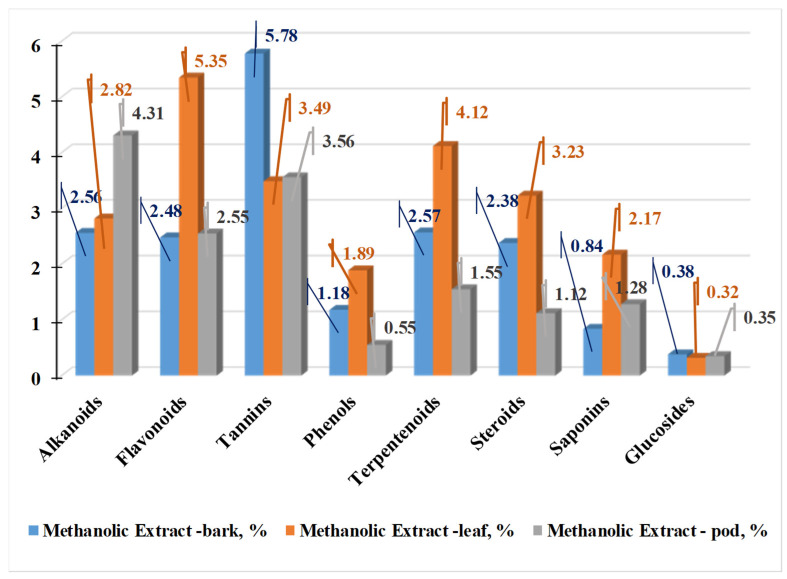
Compounds in methanol extracts of bark, leaves, and pods of *M. oleifera.*

Extracts from Madagascar have the highest flavonoid content (around 30%), followed by the ones from South Africa and Colombia. For phenolic acids, there is no such sharply defined boundary. In 2024, [49] investigated the phytochemical composition of *M. oleifera* leaves, bark, and pods in polar solvents. Figure 7, Figure 8 and Figure 9 present the results of their studies using water, ethanol, and methanol extracts.

**Figure 10:**
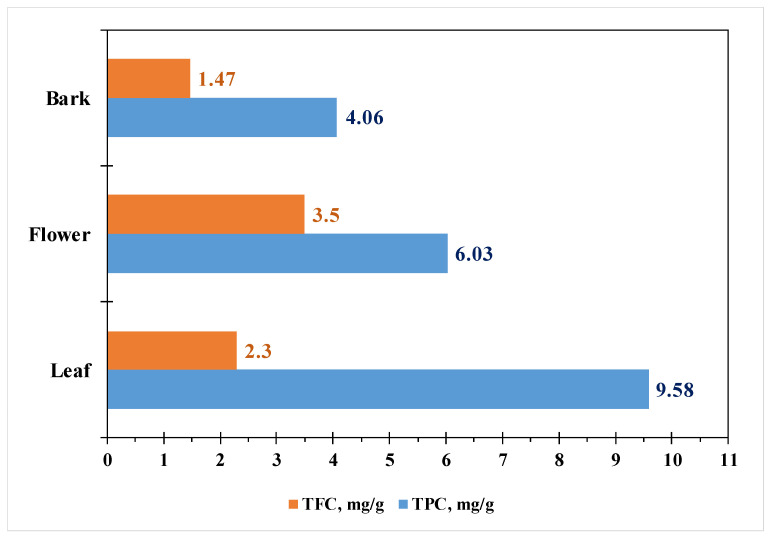
Total phenolic and flavonoid content of different parts of *M. oleifera*.

The study by [49] shows that the concentration of secondary metabolites changes when different solvents are used. The aqueous extract from all parts of the plant yields the maximum amounts of alkaloids, tannins, phenols, terpenoids, and sterols. Plant pod extracts contain the most alkaloids, while bark extracts contain the most tannins. All the aforementioned compounds have the highest content in the extracts from the leaves. Lu et al. [50] demonstrate that anthocyanin concentration is highest in the bark (52.80 mg/g), followed by the flowers (40.90 mg/g) and the leaves (9.40 mg/g) [38]. Figure 10 presents the phenolic and flavonoid content of *M. Oleifera* leaves, flowers, and bark. The flowers have the highest antioxidant activity (AOA) using the DPPH method (405 µg/mL), followed by the leaves (610 µg/mL) and finally the bark (890 µg/mL).

Flavonoids are primarily found in the flowers and leaves of the plant. The main flavonoids in the leaves are myricetin, rutin, apigenin, quercetin, and kaempferol [51]. These compounds contribute significantly to the antioxidant activity of the extracts. Wang et al. discovered that using a reflux condenser to extract these compounds from leaves at a subcritical temperature could increase the extraction by 26.7% [52]. The seeds are rich in α-tocopherols, γ-tocopherols, and δ-tocopherols [53,54]. In addition to the aforementioned chemicals, the leaves also contain linolenic acid, myristic acid, oleic acid, palmitic acid, α-linolenic acid, and α-linolenic acid [48]. Other authors expand the fatty acid composition to include eicosanoid acid, erucic acid, and palmitic acid [53].

## 6. Therapeutic Potential of *M. oleifera* Lam.

### 6.1. Antioxidant Activity

#### 6.1.1. In Vitro Antioxidant Activity

**Figure 11:**
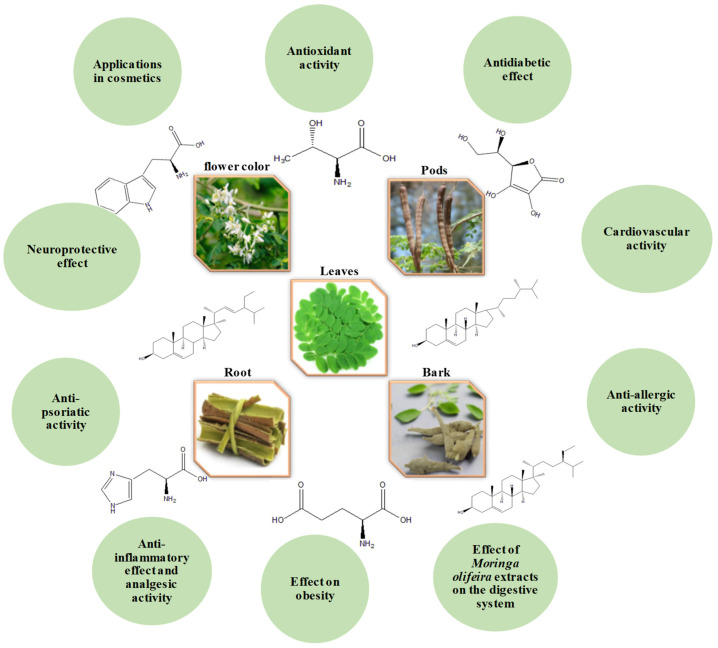
Beneficial effects of the plant *M. oleifera* on the human body.

*Moringa oleifera* contains a wide range of antioxidant compounds, including flavonoids, phenolic acids, tannins, carbamates, and isothiocyanates [24,55,56]. Its polyphenol content has been reported to surpass that of commonly consumed vegetables such as spinach, broccoli, and cabbage [57]. Numerous in vitro studies support the antioxidant potential of extracts derived from various plant parts, with the highest activity consistently attributed to the leaves due to their rich polyphenolic and flavonoid content. Lower antioxidant activity has been observed in the stems, roots, and seeds [58,59,60,61,62,63]. However, variations in assay methods, extraction solvents, and growing conditions often influence reported results [61] (Figure 11).

Table 6 presents a comparative overview of antioxidant activity across different plant parts and solvent systems. Leaf extracts showed the strongest antioxidant potential in all tested assays, particularly when extracted with ethyl acetate (IC_50_ = 5.72 µg/mL) or crude methanol (35.42 µg/mL). In contrast, extracts from stems and roots showed weaker activity, and those from seeds were generally the least effective. Solvent polarity also played a crucial role: medium-polar solvents like ethyl acetate were highly effective at concentrating flavonoids, whereas methanol-based extracts, while broader in scope, were often less potent. Notably, dry leaves demonstrated strong antioxidant performance in both FRAP and ORAC assays, suggesting their suitability for nutraceutical applications. Nonetheless, differences in extraction protocols and assay designs caution against direct cross-study comparisons.

Collectively, these findings underscore the prominence of *M. oleifera* leaves as the most antioxidant-rich plant part and highlight the critical importance of solvent selection in maximizing extract potency. Methodological discrepancies, such as differences in plant origin and assay calibration, must be considered when interpreting absolute values.

#### 6.1.2. Cellular and In Vivo Evidence for Antioxidant Activity

Oxidative stress, characterized by the excessive accumulation of reactive oxygen species (ROS), disrupts cellular homeostasis and contributes to cellular damage and death. Studies have shown that *M. oleifera* leaf extract enhances antioxidant defenses both in vitro and in vivo. In a mouse C_2_C_12_ myoblast model treated with hydrogen peroxide (H_2_O_2_), leaf extract significantly increased total antioxidant capacity and improved the redox status by elevating the ratio between reduced glutathione (GSH) and oxidized glutathione (GSSG), a key marker of intracellular redox balance. This intervention also enhanced cell viability and regenerative capacity following oxidative injury [38].

Flavonoids such as quercetin are largely responsible for these effects. In A549 lung epithelial cells stimulated with lipopolysaccharide (LPS), quercetin reduced pro-inflammatory cytokines (TNF-α, IL-1, IL-6), suppressed NF-κB nuclear translocation, and downregulated NOX2 expression—a major ROS-generating enzyme—at both mRNA and protein levels [66].

In vivo, the administration of aqueous leaf extract mitigated Abamectin-induced oxidative disturbances in mice, restoring acetylcholinesterase and glutathione *S*-transferase activities in the brain and elevating reduced GSH levels in both brain tissue and erythrocytes [67].

### 6.2. Anti-Inflammatory Activity

Numerous in vitro and in vivo studies confirm the anti-inflammatory activity of *M. oleifera*. The anti-inflammatory effects of the plant are associated with the content of flavonoids (quercetin, kaempferol), phenolic acids (e.g., chlorogenic acid), isothiocyanates, tannins, and saponins. A detailed description of the phytochemicals contained in the plant and the mechanisms through which they inhibit inflammation has been presented by [11]. The biologically active compounds suppress inflammatory processes through several mechanisms. Many of the antioxidant compounds in the plant, such as quercetin and kaempferol, also contribute to its anti-inflammatory effects, highlighting the interconnected nature of these activities. The above-mentioned flavonoids inhibit key pro-inflammatory enzymes like cyclooxygenase (COX) and lipoxygenase and downregulate cytokines such as IL-1β, IL-6, PGE_2_, and TNF-α in RAW 264.7 macrophages [11]. β-sitosterol (BSS) is a widespread phytosterol found in many plant species, its anti-inflammatory activity has been investigated in formulations containing *M. oleifera*-derived BSS. In vitro studies demonstrate that BSS nanoparticles can suppress the secretion of inflammatory mediators such as TNF-α, IL-1β, and ROS in keratinocytes and macrophages [68].

A hydroethanolic extract of *M. oleifera* flowers reduced NO, PGE_2_, and cytokine production in LPS-stimulated macrophages, primarily through inhibition of the NF-κB signaling pathway [69,70]. Glucosinolates and their hydrolysis products, such as moringin, also contribute significantly to the anti-inflammatory effects [71,72]. Leaf tea preparations rich in moringin were shown to inhibit inducible nitric oxide synthase (iNOS) expression in LPS-stimulated mouse macrophages [73].

The anti-inflammatory potential of *M. oleifera* has been widely validated in animal models. Topical application of MIC (an isothiocyanate from seeds) and MSE (seed extract) in a TPA-induced mouse ear edema model significantly reduced pro-inflammatory cytokines, including IL-6, monocyte chemoattractant protein-1 (MCP-1), and keratinocyte chemoattractant (KC) [74].

In rat paw edema models, both aqueous and ethanolic leaf extracts reduced inflammation induced by carrageenan and egg albumin, showing efficacy comparable to ibuprofen and diclofenac [75,76]. Ezeamuzie et al. initially reported a 35–45% reduction in paw edema using root and leaf extracts [75].

In a streptozotocin-induced diabetic rat model, treatment with methanolic leaf extract led to a marked decrease in hepatic and renal levels of IL-1, IL-12, IL-18, and NF-κB, while also exhibiting antioxidant and anti-apoptotic effects [77].

Additional research demonstrated a bronchodilator and anti-asthmatic effect of methanolic leaf extract in ovalbumin-sensitized guinea pigs, as evidenced by a reduction in white blood cell count and histamine levels in lung tissue [78].

Beyond inflammation, *M. oleifera* extracts have shown analgesic effects, demonstrated using the hot plate and acetic acid writhing methods. Extracts from leaves, seeds, bark, and roots have been effective, with some studies suggesting dose-dependent anti-migraine potential [79,80,81].

These in vivo findings highlight the multi-faceted anti-inflammatory potential of *M. oleifera* extracts, especially from leaves and seeds. A summary of selected in vivo studies, extract types, and their effects is presented in Table 7.

### 6.3. Antimicrobial (Antibacterial, Antifungal) Activity

Among the different parts of *M. oleifera*, the leaves and seeds exhibit the highest antibacterial efficacy. Their extracts are active against a broad spectrum of Gram-positive and Gram-negative bacteria, underscoring the plant’s potential as a natural source of antibacterial agents. These antimicrobial effects are mediated through multiple mechanisms, including disruption of bacterial cell membranes, inhibition of key metabolic enzymes, interference with quorum sensing and biofilm formation, and induction of oxidative stress [61,82,83,84,85,86].

*Moringa* seed powder has demonstrated antibacterial activity against several pathogens, with the strongest effects observed against *Salmonella* spp. [87]. Flavonoids such as kaempferol and quercetin, identified in seed extracts, contribute significantly to this activity: kaempferol inhibited *Klebsiella pneumoniae*, *Pseudomonas aeruginosa*, and *Streptococcus pneumoniae*, while quercetin was most effective against *Staphylococcus aureus* [84]. Additionally, isothiocyanates isolated from seeds exhibited strong activity against Gram-positive bacteria, including *S. aureus*, *S. epidermidis*, and *Bacillus subtilis* [88]. *Moringa* extracts have also been shown to inhibit biofilm formation, a critical factor in persistent infections, particularly those involving *S. aureus* [86].

The antibacterial efficacy of *M. oleifera* can be further enhanced through combination with conventional antibiotics. Studies have demonstrated synergistic effects, particularly with ethanol leaf extracts. Ashraf et al. [89] reported that combining *M. oleifera* extracts with antibiotics such as gentamicin, ampicillin, enrofloxacin, and oxytetracycline significantly improved activity against *E. coli*, *S. aureus*, and *Klebsiella* spp., highlighting its potential to combat antibiotic resistance.

Nanotechnology-based formulations of *M. oleifera* further enhance its antibacterial properties. Zinc oxide nanoparticles synthesized using *M. oleifera* leaf extract (MOL: ZnO NPs) exhibited strong inhibitory effects against *Pseudomonas* spp. and *B. cereus*, primarily through membrane disruption mechanisms [90]. Similarly, silver nanoparticles (Ag-NPs) derived from *M. oleifera* showed superior antibacterial activity compared to crude aqueous extracts, suggesting that nanoparticle delivery systems can improve the bioavailability and potency of plant-derived antimicrobials [91].

In the context of oral health, *M. oleifera* extracts from seeds, roots, and leaves have demonstrated inhibitory activity against common oral pathogens such as *Streptococcus mutans* and *S. aureus*. Ethanol leaf extracts showed the greatest efficacy and have been incorporated into formulations like toothpaste and mouthwash [92]. Aqueous extracts have also been effective against bacteria associated with orthopedic wound infections, supporting their potential use in topical antimicrobial applications [93]. Clinically, *M. oleifera* bark extract significantly improved outcomes in patients with urinary tract infections, with a 67% recovery rate compared to 47% in the group receiving standard medications, highlighting its antibacterial and anti-inflammatory therapeutic potential [94].

Overall, the antibacterial activity of *M. oleifera* is well-supported by in vitro and clinical evidence. Its phytochemicals, particularly flavonoids and isothiocyanates, exert potent, multi-target effects against many pathogens. The plant’s ability to enhance conventional antibiotic efficacy, inhibit biofilms, and perform comparably to standard therapies in clinical settings positions it as a promising candidate for the development of plant-based antibacterial formulations. Continued investigation into standardized extraction methods and bioactive synergy will be essential to advance its integration into medical and pharmaceutical applications.

*M. oleifera* has demonstrated promising antifungal activity against a broad spectrum of human and phytopathogenic fungi (Table 8). Extracts from various plant parts, particularly the leaves, seeds, and oils, have shown efficacy against dermatophytes (e.g., *Trichophyton*, *Microsporum*, *Epidermophyton* spp.), yeasts (e.g., *Candida* spp., *Saccharomyces cerevisiae*), and molds (*F. solani*, *F. oxysporum*).

Aqueous and ethanolic leaf extracts inhibited *C. tropicalis* and *S. cerevisiae*, although early studies noted limited activity against *C. albicans* [95]. However, more recent investigations report significant antifungal effects against *C. albicans*, with ethanolic extracts yielding larger inhibition zones than aqueous preparations [96]. In vitro tests have also confirmed the efficacy of ethanol extracts against dermatophytes such as *T. rubrum*, *T. mentagrophytes*, *E. floccosum*, and *M. canis* [97].

*M. oleifera* oil has shown antifungal activity against multiple *Candida* species (e.g., *C. albicans*, *C. dubliniensis*, *C. glabrata*, *C. kefyr*, *C. krusei*, *C. lusitaniae*) isolated from stool samples of autistic children, suggesting therapeutic potential for gastrointestinal candidiasis [98]. Additionally, extracts from various plant parts have displayed fungicidal effects against several phytopathogenic fungi, underscoring potential applications as natural biofungicides in agriculture [99].

A notable mechanism of antifungal action involves chitin-binding proteins (CBPs) isolated from *M. oleifera* seeds. Mo-CBP3, for example, inhibited the growth of *Fusarium solani*, *F. oxysporum*, *Colletotrichum musae*, and *C. gloeosporioides* in vitro [100]. This protein demonstrated both fungistatic and fungicidal effects against *F. solani*, depending on the concentration used [101]. Additional CBPs, including Mo-CBP2 and Mo-CBP4, have shown potent activity against *Candida* spp. and dermatophytes. Mo-CBP4, in particular, reduced the severity of *T. mentagrophytes* infection in a murine dermatophytosis model by increasing membrane permeability, inducing reactive oxygen species (ROS), and damaging fungal cell walls [102,103].

Collectively, these findings highlight the potential of *M. oleifera* extracts and seed-derived CBPs as novel antifungal agents for both medical and agricultural applications.

**Table 8:** Antibacterial and antifungal effects of *M. oleifera*.

Type	Part of the Plant	Dose	References
Antibacterial effects			
*S. typhi*	Leaves (ethanolic extract)	800 mg/mL	[83]
*S. aureus*, *E. faecalis*, *B. subtilis*, *S. typhi*, *E. coli*	Leaves (petroleum ether extract)	62.5, 125, 250, 10,000 μg	[59]
*S. aureus*, *B. subtilis*, *E. coli*, *P. aeruginosa*	Seeds (aqueous and methanolic extracts)	EC 5, 10, 20, 40%	[83]
*S. aureus*, *V. parahaemolyticus*, *E. faecalis*, *A. caviae*	Leaves (aqueous and ethanolic extracts)	400 μL (20 g/180 mL)	[85]
*Salmonella* sp., *E. coli*, *S. aureus*	Seed (powder)	0.017 g/mL	[87]
*E. coli*, *K. pneumoniae*, *P. aeruginosa*, *S. pneumoniae*, *S. aureus*.	Seeds (methanolic extract)	2, 4, 6 mg/mL	[84]
*S. aureus*, *S. epidermidis*, *B. subtilis*	Isothiocyanates isolated from seeds	1, 10 mg/mL	[88]
*S. aureus*	Leaves (aqueous and saline extracts)	100, 200 μg/mL	[86]
*K. pneumoniae*, *E. coli*, *S. aureus*.	Leaves (ethanolic extract)	1000–3906 mg/mL	[89]
*L. monocytogenes*	Moringin, isolated from seeds	0.124 mg/mL	[104]
*E. coli*, *S. sciuri*, *S. aureus*, *S. typhi*, *S. enterica*, *P. aeruginosa*	Leaves (ethanolic extract, aqueous extract)	0.04–0.42 mg/mL 0.03–0.33 mg/mL	[91]
*S. aureus*, *S. mutans*	Seeds, roots, leaves (ethanolic, acetate, ethyl-acetate extracts)	400 mg/mL	[92]
*E. coli*, *S. typhi*, *S. aureus*, *Enterococcus* sp., *P. aeruginosa*	Leaves (aqueous and methanolic extracts)	30 mg/mL	[93]
Antifungal effects			
Yeasts (Candida species, etc.)			
*C. albicans*	Leaves (ethanolic and aqueous extracts)	100, 200, 300, 400, 500 µg/mL	[96]
*C. albicans*, *C. dulblinesis*, *C. glabarata*, *C. kefyr*, *C. krusei*, *C. lusitania.*	Seeds (oil)	1.0%	[98]
*C. albicans*, *C. parapsilosis*, *C. krusei*, *C. tropicalis.*	Seeds (purified protein, Mo-CBP_2_)	0.32 mg/g	[102]
Dermatophytes			
*T. rubrum*, *T. mentagrophytes*, *E. xoccosum*, *M. canis*	Leaves (crude essential oil) (ethanolic extract) Seed extract	0.2. 0.4, 0.6, 1.6 mg/mL 2.5 mg/mL 2.5 mg/mL	[97]
*E. floccosum* and *T. rubrum*	Isothiocyanates isolated from seed extract	1, 10 mg/mL	[88]
Phytopathogenic fungi			
*Fusarium solani*, *F. oxysporum*, *C. musae* and *C. gloesporioides*	Protein Mo-CBP_3_ isolated from seeds	0.05 mg/mL	[100,101]
*T. mentagrophytes*	Protein Mo-CBP_4_ isolated from seeds	5, 10, 20 mg/g	[103]

### 6.4. Antiviral Activity

*M. oleifera* has demonstrated promising antiviral activity in both in vitro and in vivo studies, attributed to its rich content of bioactive phytochemicals (Table 9). Extracts and isolated compounds from various parts of the plant have shown inhibitory effects against a wide range of viruses, including Hepatitis B virus (HBV), Influenza A virus subtype H1N1 (A/H1N1), Herpes Simplex Virus Type 1 (HSV-1), SARS-CoV-2, Foot and Mouth Disease Virus (FMDV), and Newcastle Disease Virus (NDV).

Aqueous leaf extract inhibited HBV replication in Huh7 liver cells expressing HBV genotypes, demonstrating its antiviral potential at the cellular level [105]. In vivo, oral administration of aqueous extract significantly limited the development of skin lesions in HSV-1-infected mice and enhanced immune response by increasing interferon-gamma (IFN-γ) production [106].

In the context of SARS-CoV-2, *M. oleifera* has attracted substantial attention due to its phytochemical diversity. Computational studies have identified 22 bioactive compounds with predicted affinity for viral targets. Flavonoids such as rutin, myricetin, quercetin, apigenin, and especially ellagic acid demonstrated strong binding to the spike (S) protein of SARS-CoV-2, suggesting potential to block viral entry [107]. Additionally, kaempferol-3-*O*-rutinoside and vitexin formed stable complexes with the main protease (Mpro), a critical enzyme in viral replication [108]. Similar inhibitory effects were observed for quercetin-3-rhamnoside, myricetin-3-rutinoside, and rutin [109].

These findings support the hypothesis that *M. oleifera* extracts may interfere with key stages of viral infection and replication. Moreover, the water-soluble nature of many of these compounds favors intestinal absorption and possibly even passage through the blood-brain barrier—enhancing their potential as orally administered antiviral agents.

**Table 9:** Antiviral activity of *M. oleifera* extracts against various viral pathogens.

Virus Type	Plant Part (Extract Type)	Dose/EC_50_/EC_90_	References
Influenza Virus (H1N1)	Seeds (ethanolic)	EC_50_ = 1.27 µM; EC_90_ = 5.30 µM	[105]
Herpes Simplex Virus Type 1 (HSV-1)	Leaves	300 mg/kg (in vivo)	[106]
Hepatitis B Virus (HBV)	Leaves (aqueous)	30, 45, and 60 µg/mL	[105]
Foot and Mouth Disease Virus (FMDV)	Leaves (ethanolic)	1.6, 6.12, 25, 50, 100, 200 µg/mL	[110]
Newcastle Disease Virus (NDV)	Leaves (methanolic)	200 mg/kg (in vivo)	[111]

### 6.5. Anticancer Effects

*M. oleifera* exhibits promising anticancer potential due to its rich composition of bioactive compounds that modulate oxidative stress, promote apoptosis, and interfere with tumor-promoting signaling pathways.

#### 6.5.1. In Vitro and Mechanistic Evidence

Numerous in vitro studies have demonstrated the cytotoxic effects of *M. oleifera* extracts against a variety of cancer cell lines, including cervical (HeLa) [112], leukemia and hepatocarcinoma (HepG2) [113], colon [114], lung adenocarcinoma (A549) [115], breast and colorectal cancer [116].

Bioactive fractions from leaves, seeds, and bark have shown the ability to trigger apoptosis, modulate oxidative balance, and suppress tumor cell proliferation. For instance, ethyl acetate leaf extract rich in phenolics inhibited melanoma cell growth via caspase-dependent and -independent pathways [117], while an alkaloid extract upregulated caspase-3 and -9 in A549 lung cells [118]. In PC3 prostate cancer cells, methanolic leaf extract enhanced caspase-3 activity by downregulating the Hedgehog pathway [119].

*M. oleifera* compounds such as quercetin, kaempferol, and benzyl isothiocyanate have been shown to induce cell cycle arrest by regulating cyclins and cyclin-dependent kinases, thereby limiting cancer cell division [114,118,120,121].

Moreover, inhibition of key oncogenic signaling cascades—such as NF-κB, PI3K/AKT, JAK2/STAT3, and MAPK—has been reported following treatment with *M. oleifera* extracts, highlighting their role in suppressing tumor growth and metastasis [116,118,120]

Antioxidant-rich compounds in *M. oleifera* also contribute to DNA protection by reducing oxidative damage. Phenolic-rich leaf extracts counteracted DNA damage in tumor KB cells [122,123], while aqueous extracts induced oxidative stress and cell cycle arrest (sub-G0 phase) in colon cancer lines, demonstrating a dual role depending on the cellular context [114].

There is also emerging evidence of epigenetic modulation. Moringa isothiocyanate (MIC-1) was shown to reverse aberrant DNA CpG methylation during carcinogenic transformation in JB6 cells, suggesting its potential to influence gene expression patterns linked to tumorigenesis [124].

#### 6.5.2. In Vivo Studies and Cancer Chemopreventive Potential

In vivo models further support *M. oleifera*’s anticancer potential. A flower-derived trypsin inhibitor (MoFTI) reduced tumor growth and angiogenesis in a murine sarcoma model at doses of 15 or 30 mg/kg [125]. In a colon cancer mouse model, dietary supplementation with boiled *M. oleifera* pods (1.5–6.0%) over 15 weeks significantly reduced tumor incidence, with 3% being the most effective dose [124].

In a breast cancer model, *M. oleifera* extract mitigatedoxorubicin-induced toxicity by reducing inflammation and oxidative stress, demonstrating a protective role during chemotherapy [126,127]. However, combination treatment with seed extract and chemotherapy in triple-negative breast cancer unexpectedly led to the upregulation of pro-angiogenic genes, indicating that therapeutic interactions may vary depending on disease context and treatment strategy [127].

To date, no clinical trials have confirmed the anticancer efficacy of *M. oleifera* in humans, and further translational research is needed to assess its safety, bioavailability, and potential synergistic effects with conventional therapies.

### 6.6. Hepatoprotective Effects

*M. oleifera* has demonstrated significant hepatoprotective activity in various animal models of chemically induced liver injury. Experimental studies using toxic agents such as carbon tetrachloride (CCl_4_), bisphenol-A, cadmium, paracetamol, acetaminophen, and lead consistently show that *M. oleifera* extracts can prevent or mitigate liver damage through both antioxidant and anti-inflammatory mechanisms.

Administration of *M. oleifera* leaf extracts led to a marked reduction in serum liver enzymes—alanine transaminase (ALT), aspartate aminotransferase (AST), and alkaline phosphatase (ALP)—which are biomarkers of hepatic injury. Histopathological analyses corroborated these findings, revealing improved hepatic architecture and reduced hepatocellular necrosis in treated animals [128,129].

In CCl_4_-induced liver damage, leaf extract significantly lowered levels of bilirubin, glutamate pyruvate transferase (GPT), glutamate oxaloacetate transferase (GOT), ALP, and lysosomal enzymes, with corresponding improvements in hepatocyte morphology [130]. Similarly, rats exposed to cadmium and treated with *M. oleifera* (500 mg/kg for 28 days) exhibited normalized liver enzymes and reduced lipid peroxidation [131].

Mechanistically, the hepatoprotective effects of *M. oleifera* are attributed to its ability to suppress oxidative stress, inhibit pro-inflammatory cytokines (e.g., TNF-α and IL-1β), and modulate the NF-κB signaling pathway [130,131,132,133]. For instance, aqueous leaf extract (100–200 mg/kg) significantly attenuated acetaminophen-induced hepatotoxicity by restoring hepatic glutathione levels and reducing serum transaminases, nitrite, and malondialdehyde (MDA) content [131,134]. In another study, microencapsulated *M. oleifera* oil reduced hepatic oxidative stress and inflammation in rats fed a high-fat diet. Additionally, the hepatoprotective action of the leaf extract in rats with levofloxacin-induced liver damage was linked to its antioxidant properties, as evidenced by the normalization of liver enzymes (ALT, AST, GGT) and restoration of antioxidant balance [135].

### 6.7. Antidiabetic Effects

*M. oleifera* has demonstrated antidiabetic potential in both experimental animal models and human studies. Extracts from its leaves and seeds have been shown to reduce blood glucose levels, improve lipid profiles, and mitigate diabetes-induced tissue damage. These effects are attributed to its rich content of bioactive compounds with antioxidant and anti-inflammatory properties, which modulate key biochemical pathways involved in glucose and lipid metabolism [136,137].

In vitro studies indicate that *M. oleifera* leaf extracts inhibit carbohydrate-digesting enzymes such as α-glucosidase and pancreatic lipase, thereby reducing glucose absorption and improving insulin sensitivity in adipocyte models (3T3-L1 cells) [138]. The inhibition of α-amylase and α-glucosidase enzymes is a recognized strategy to reduce postprandial hyperglycemia, especially in patients with type 2 diabetes [139].

In vivo, administration of *M. oleifera* leaf extract (100 or 200 mg/kg orally) for eight weeks in streptozotocin (STZ)-induced diabetic rats significantly reduced blood glucose and oxidative stress markers. It also improved renal histology and downregulated pro-inflammatory markers such as TGF-β1 and collagen IV expression in diabetic nephropathy [140]. Aljazzaf et al. further showed that combined methanolic extracts of leaves and seeds (500 mg/kg) administered for 13 months to alloxan-induced diabetic mice improved antioxidant status, lipid profiles, and histopathological outcomes in liver and kidney tissues [141].

The stem extract also exhibited protective effects on pancreatic β-cells by modulating oxidative stress and cell signaling pathways, reinforcing the therapeutic relevance of non-leaf tissues [142].

Clinical studies support these findings. In postmenopausal women, supplementation with *M. oleifera* leaf powder over three months led to a reduction in fasting blood glucose levels [143]. A randomized placebo-controlled trial demonstrated that prediabetic individuals taking dry leaf powder capsules exhibited significant decreases in fasting glucose and HbA1c levels compared to controls [144]. In obese individuals with type 2 diabetes, 40-day supplementation with leaf powder reduced serum glucose and LDL cholesterol levels [145].

Together, these results suggest that *M. oleifera* offers promising therapeutic value in diabetes management, potentially serving as an adjunct to conventional treatments.

### 6.8. Cardiovascular Effects

The cardioprotective potential of *M. oleifera* has been increasingly supported by experimental evidence, much of which is linked to its antioxidant, hypoglycemic, and hypolipidemic properties. These effects are attributed to a variety of phytochemicals—including quercetin, apigenin, and lupeol—known to modulate oxidative stress and vascular function.

In vivo studies have shown that supplementation with *M. oleifera* seed powder exerts antihypertensive effects in spontaneously hypertensive rats, with improvements in blood pressure regulation and cardiac function [146,147]. Similarly, in isoproterenol-induced myocardial injury in Wistar rats, chronic administration of leaf extracts normalized serum biochemical parameters, reduced lipid peroxidation, and mitigated histopathological damage to cardiac tissue [148].

Cardioprotective effects were also observed in a model of potassium bromate-induced cardiac dysfunction, where treatment with aqueous leaf extract restored serum levels of AST, ALT, and ALP, markers of cardiac and hepatic stress [149].

Mechanistic insights suggest that the cardiodepressive effects of *M. oleifera* may involve the activation of muscarinic M2 receptors, leading to reduced cardiac contractility and enhanced vasodilation, ultimately lowering peripheral resistance and blood pressure [150].

Together, these findings support the potential application of *M. oleifera* extracts as adjunct agents in managing cardiovascular conditions such as hypertension, myocardial injury, and ischemic heart disease.

### 6.9. Neuroprotective Effects

Neurodegenerative disorders, including Alzheimer’s disease, Parkinson’s disease, and dementia, are commonly associated with progressive neuronal loss caused by oxidative stress, neuroinflammation, and mitochondrial dysfunction. Emerging evidence from in vitro and in vivo studies suggests that *M. oleifera* exhibits promising neuroprotective potential against such conditions, as well as acute injuries like stroke and neurotoxic damage [151].

These effects are largely attributed to the high levels of polyphenolic compounds in *M. oleifera*, which exhibit potent antioxidant activity. In human neuroblastoma SH-SY5Y cells, leaf extract significantly reduced oxidative stress by lowering lipid peroxidation, enhancing endogenous antioxidant enzyme activity, and preserving mitochondrial function, all while maintaining low cytotoxicity [152,153]. In vivo, moringa tree extracts protected neural tissue in models of ischemic stroke and pesticide-induced neurotoxicity [151,152,153,154,155,156].

Seed extracts, rich in glucosinolates such as niazimicin (a thiocarbamate glycoside), have demonstrated protective effects in neurodegenerative models. In rotenone-induced Parkinson’s disease in mice, moringa seed extract reduced motor dysfunction and histologically preserved dopaminergic neurons [157]. Similarly, niazimicin-containing ethanol seed fractions alleviated oxidative damage and behavioral deficits in an aluminum chloride-induced dementia model [158].

In a rat model of hyperhomocysteinemia-induced Alzheimer’s disease, methanolic leaf extract administered at 400 mg/kg/day improved outcomes both as a preventive and curative agent. It reduced oxidative stress, inhibited tau hyperphosphorylation, lowered amyloid-β production, and mitigated neurodegenerative pathology, supporting a multifaceted neuroprotective mechanism involving antioxidative and anti-inflammatory pathways [159].

Beyond biochemical and histological protection, *M. oleifera* leaf extracts have also been shown to improve cognitive performance. In several models—including age-related dementia, scopolamine-induced amnesia, and maternal protein deficiency moringa supplementation improved learning, memory, and spatial navigation abilities [155,160,161,162].

### 6.10. Gastrointestinal Protective Effects

*M. oleifera* demonstrates notable gastroprotective properties. In a rat model of bisphenol A-induced gastric ulcers, oral administration of leaf extract (200 mg/kg for four weeks) significantly reduced ulceration, accompanied by potent antioxidant, anti-apoptotic, and anti-inflammatory effects [163]. Aqueous seed extract (2000 mg/kg for 14 days) also protected against indomethacin-induced gastric ulcers, though its effect was not as strong as that of the reference drug cimetidine [164]. In a mouse model of ulcerative colitis, oral administration of a polysaccharide isolated from *M. oleifera* alleviated intestinal damage and suppressed the release of pro-inflammatory cytokines [165]. The antibacterial, anti-inflammatory, and ulcer-protective properties of *M. oleifera* contribute to its anti-diarrheal effect, which has been documented in models of bacterial gastroenteritis [166,167].

### 6.11. Anti-Obesity Effects

Obesity is a chronic metabolic condition marked by excessive fat accumulation and is closely associated with an increased risk of type 2 diabetes, cardiovascular disease, stroke, and certain cancers. *M. oleifera* has been extensively investigated for its anti-obesity effects, which are primarily attributed to its rich profile of bioactive compounds with antioxidant, anti-inflammatory, and lipid-lowering properties. These compounds exert their effects by improving lipid metabolism, reducing adiposity, modulating hormone levels, and enhancing the expression of genes involved in fat oxidation.

In high-fat diet (HFD)-induced obese rats, oral administration of *M. oleifera* methanolic extract (200 and 400 mg/kg for 12 weeks) significantly reduced body weight, total cholesterol, and triglyceride levels, while increasing HDL-C and antioxidant enzyme activity [168]. Similar results were reported in HFD-obese mice, where fermented *M. oleifera* extract reduced hepatic lipid accumulation and downregulated pro-inflammatory cytokine mRNA expression [169]. Additionally, ethanolic leaf extract (5.6 or 11.2 mg/20 g body weight/day for 7 weeks) prevented hematological disturbances in HFD-fed mice, including improvements in hemoglobin levels and white blood cell profiles [170].

At the molecular level, *M. oleifera* extracts suppressed the expression of obesity-related hormones such as leptin and resistin, while increasing adiponectin levels—an effect comparable to the lipid-lowering drug Simvastatin [171]. Other studies demonstrated that *M. oleifera* ethanol extract decreased adiposity index, blood glucose, insulin resistance markers, and downregulated lipogenic enzymes like fatty acid synthase and HMG-CoA reductase. It simultaneously enhanced the expression of PPARα and MC4R, facilitating β-oxidation and reducing fat accumulation in hepatic and adipose tissues [172].

In guinea pigs fed a high-cholesterol diet, dietary inclusion of moringa leaves (2 or 3.5 g/day) significantly reduced hepatic cholesterol and triglyceride levels, inflammatory cytokines (IL-1β, IL-10, IFN-γ), and histologically evident liver steatosis, without altering plasma lipid profiles. These findings suggest modulation of hepatic lipid synthesis pathways as a key mechanism [173].

Human clinical trials, although still limited, support these findings. In a 12-week randomized controlled trial with 40 overweight hyperlipidemic subjects, supplementation with *M. oleifera* leaf powder resulted in reductions in body weight, BMI, waist circumference, blood pressure, triglycerides, and LDL cholesterol [174]. Similarly, a double-blind, placebo-controlled study in overweight women (400 mg ethanolic extract/day for 8 weeks) showed significant decreases in BMI, total cholesterol, and LDL levels compared to placebo [172].

Together, these data from preclinical and clinical studies indicate that *M. oleifera* may be a promising natural adjunct for managing obesity and associated metabolic disturbances through multi-targeted mechanisms.

### 6.12. Effects on Fertility

*M. oleifera* has been traditionally used in ethnomedicine across parts of Africa and Asia to influence reproductive function. Scientific studies have confirmed that the plant exhibits both fertility-enhancing and antifertility effects, depending on factors such as plant part, dose, extract type, sex of the subject, and physiological context.

#### 6.12.1. Fertility-Enhancing Effects

The antioxidant, anti-inflammatory, and androgen-supporting properties of *M. oleifera* contribute to improved male reproductive health. These effects are largely attributed to the plant’s bioactive constituents, which protect against oxidative damage, improve testicular architecture, and restore hormonal balance.

In animal models, *M. oleifera* leaf extract has ameliorated testicular toxicity caused by melamine, improving sperm quality, testicular weight, follicle-stimulating hormone (FSH), and testosterone levels [175]. In rats with experimentally induced cryptorchidism, aqueous extract (400–800 mg/kg, administered orally) reduced germ cell apoptosis, oxidative stress, and heat shock protein 70 expression, preserving testicular integrity [176].

In a high-fructose diet rat model, *M. oleifera* aqueous leaf extract (300 mg/kg) reversed hepatic insulin resistance, improved serum testosterone levels, and upregulated genes involved in steroidogenesis (e.g., StAR and 3β-HSD), further supporting its reproductive benefits [177].

#### 6.12.2. Antifertility and Abortifacient Effects

Conversely, *M. oleifera* has demonstrated antifertility effects, particularly in female animal models. Studies have reported abortifacient activity, impaired pregnancy outcomes, and toxic effects on offspring, particularly with seed extracts.

In pregnant Wistar rats, lipid-rich aqueous-methanol seed extracts caused hepatic and renal toxicity in offspring and restricted uterine development in females, while lipid-free extracts at doses below 300 mg/kg showed no adverse reproductive outcomes [178]. An aqueous extract (175 mg/kg) caused complete abortion in pregnant rats, reflecting its ethnomedical use in rural India as an early-stage abortifacient [179]. However, at lower doses (30 mg/kg), the same extract ameliorated postpartum depression symptoms without abortive effects, improving maternal behavior and lactation [180].

Further evidence shows that ethanol extract (250–500 mg/kg) inhibited implantation in decidualized female rats, indicating anti-implantation activity [181]. A dietary study in rabbits revealed sex-dependent hormonal modulation: while male rabbits showed improved fertility, increased FSH and LH, and enhanced semen quality, female rabbits exhibited reduced FSH, LH, and estrogen, with elevated progesterone at higher doses [182].

These findings suggest that *M. oleifera* modulates reproductive function in a complex, context-dependent manner. While its use may benefit male fertility and protect against reproductive toxins, caution is warranted regarding its use during pregnancy due to its potential abortifacient effects. Further studies are needed to clarify the safe therapeutic window and underlying mechanisms of these divergent effects.

### 6.13. Effects on Bones

In vivo and in vitro studies have demonstrated the positive effects of *M. oleifera* leaf extract on bone health, including stimulation of osteoblastic cell proliferation, bone matrix formation and mineralization, and reduction of bone resorption. In rabbit models with critical-sized mandibular bone defects, the application of *M. oleifera* leaf extract in combination with beta-tricalcium phosphate enhanced bone regeneration. Histological analyses revealed increased new bone formation and a higher number of osteoblasts in the treated groups compared to controls. [14], In the ovariectomized rat model (a postmenopausal osteoporosis model), supplementation with *M. oleifera* leaf powder significantly increased bone mineral density and improved bone microstructure. The effects were associated with the modulation of gut microbiota and the MAPK signaling pathway, suggesting a multifaceted mechanism of action [183]. A recent study identified bioactive peptides derived from *M. oleifera* leaves with osteoporosis-modulatory properties. Cellular assays showed that one of these, DPYLGK, significantly inhibits bone resorption and promotes bone formation [184]. Another in vitro study reported a biphasic dose-dependent effect of *M. oleifera* leaf extract on the growth activity of osteoblast-like SaOS-2 cells, with the lower doses (25 and 50 μg/mL) enhancing bone formation, and 100 and 200 μg/mL doses inhibiting the proliferation of the cells [185].

## 7. Toxicity Studies

The safety profile of *M. oleifera* has been widely assessed in both in vitro and in vivo models to evaluate potential cytotoxicity and systemic toxicity associated with various extracts. While most studies indicate that *M. oleifera* is relatively safe at moderate doses, high concentrations or prolonged use may lead to toxicological effects, depending on the extract type, dose, and biological system used.

### 7.1. In Vitro Cytotoxicity

Several in vitro studies have examined the cytotoxic effects of *M. oleifera* extracts on cell lines:

- Methanolic leaf extract applied to HBF4 cells for 24 h caused noticeable changes at doses ≥1000 μg/mL, including increased cell size. The concentration affecting viability was ≥700 μg/mL [186].
- Aqueous leaf extract showed cytotoxic effects on A549 lung cancer cells, with 30% and 15% cell death at 400 μg/mL and 500 μg/mL, respectively [187].
- Essential oil from seeds tested on HeLa, HepG2, MCF-7, Caco-2, and L929 cell lines (0.15–1 mg/mL) showed the highest cytotoxicity in HeLa, HepG2, and MCF-7 cells, suggesting a dose- and cell type-specific toxicity [170].

### 7.2. In Vivo Toxicity in Animal Models

Zhang et al. administered aqueous-methanol leaf extract (2000 mg/kg) to Wistar rats. Blood markers (ALT, AST, total bilirubin) did not indicate acute liver damage, suggesting the lethal dose (LD_50_) exceeds 2000 mg/kg in females [188].

Adedapo et al. found that aqueous leaf extract (1600 mg/100 g) lowered serum albumin levels in rats, indicating potential hepatic stress [189]. Subacute toxicity tests with doses of 250, 500, and 1500 mg/kg showed an LD_50_ value of approximately 1585 mg/kg [190]. Asiedu-Gyekye et al. tested doses up to 5000 mg/kg and advised limiting daily intake to under 70 g of the extract to avoid the potential accumulation of toxic macro- and microelements [191].

Ajibade et al. reported no mortality at 3000 mg/100 g body weight, but acute toxicity occurred at 4000 mg/kg, with deaths recorded at 5000 mg/kg [192].

### 7.3. Safety Considerations for Human Use

Despite high-dose toxicity in animal models, *M. oleifera* extracts (particularly fruits and leaves) have shown favorable safety profiles in mutagenicity and genotoxicity assessments. Siddiqui et al. reported that fruit extracts were non-mutagenic and safe for potential use in anticancer formulations based on toxicity assays in liver cancer cells [193].

## 8. *Moringa oleifera* and Its Application in Dietary Supplements

### 8.1. Commercial Use in Dietary Supplements

Due to its rich profile of bioactive compounds, including flavonoids, phenolic acids, isothiocyanates, vitamins, and minerals, *M. oleifera* has become increasingly popular in the formulation of dietary supplements (Table 10). These products are marketed globally for their diverse health-promoting properties, ranging from antioxidant and anti-inflammatory effects to blood sugar regulation and immune system support. Supplements typically utilize leaf extracts, but preparations from seeds, bark, root, and fruit are also available, each offering specific therapeutic benefits depending on the extract type and composition.

### 8.2. Applications in Functional Foods and Nutritional Enrichment

*Moringa oleifera* is widely utilized in the development of functional foods due to its exceptional nutritional value. In African and Asian cuisines, various parts of the plant are consumed: the pods and seeds are cooked in stews, the leaves are brewed as tea or added to soups, and the roots are used as a spice with a horseradish-like flavor.

In Europe, particularly in Bulgaria, powdered *M. oleifera* leaves are becoming increasingly popular as a dietary supplement. They are incorporated into smoothies, juices, and cooked dishes to enhance micronutrient intake and support immune function. The dried leaf powder is also used for its content of essential fatty acids and bioactive polyphenols.

Experimental applications in food technology have demonstrated that incorporating *M. oleifera* into staple foods can enhance their nutritional quality. For example, adding moringa seed powder to wheat bread increases its protein, carbohydrate, and vitamin A content, while also extending shelf life by reducing fungal contamination [26,194]. However, while moringa enrichment improves the fiber and mineral content of baked goods such as biscuits and muffins, it may also affect organoleptic properties, altering taste, color, texture, and vitamin retention [30,195,196].

## 9. Cosmetic Applications

*M. oleifera* is increasingly used in cosmetic formulations due to its rich profile of bioactive compounds with antioxidant, anti-inflammatory, and anti-aging properties. The plant’s seed oil is particularly valued for its high content of essential fatty acids, including oleic, linoleic, and palmitic acids, which help nourish and hydrate the skin. Leaf extracts are also incorporated for their antioxidant polyphenols and vitamins (A, C, and E), which protect the skin from oxidative damage and environmental stressors.

Isothiocyanates derived from *M. oleifera* (MITC) have demonstrated potential in anti-aging applications. When encapsulated in nanoliposomes, MITC showed increased skin permeability (up to 71.4%), enhancing dermal absorption and photoprotection [197]. Kumalaningsih and colleagues developed a UV-protective whitening cream combining seed oil, leaf extract, and red rice-derived zinc oxide (ZnO), offering both antioxidant protection and UV-blocking activity [198].

*M. oleifera* extracts and oils from seeds, leaves, and other parts have been approved by the European Commission’s COSING database (2020) for use in cosmetic products. These include facial cleansers, micellar water, moisturizers, body lotions, eye creams, and anti-aging serums manufactured globally in Europe, North America, and Asia (Table 11).

## 10. Preparation of Moringa Formulations

*M. oleifera* formulations include granules, tablets, capsules, topical applications, and plant extracts incorporated into nanoparticles. Tablets commonly utilize binding agents such as corn starch, gelatin, and microcrystalline cellulose. Gelatin is the most suitable binding agent for the tablets, providing the lowest brittleness, appropriate strength, and easy disintegration.

Many components in plant extracts have poor solubility and low bioavailability. To address these challenges, the literature includes various modern formulations. To improve the water solubility of isothiocyanates, modified amphiphilic hyaluronic acid and ceramide nanoliposomes have been used to enhance the permeation ability of the substance through the skin [205]. Studies have also reported coating gelatin nanoparticles with *M. oleifera* extracts to enhance repellent activity [170]. Silver nanoparticles combined with leaf extract have shown anticancer activity against HTC116 and SW480 cancer cells [206]. For external applications intended to promote rapid wound healing, a hydrogel containing a hexane extract from the plant’s seeds has been recommended. Research indicates that tablets formulated with *M. oleifera* gum facilitate 91% drug release, making them a promising carrier for colon-targeted drug delivery [207].

## 11. Existing Patents on Compositions with Extracts from Different Parts of *M. oleifera*

In 2009, Jiaheng Zhang patented a formulation derived from the leaves and seeds of *M. oleifera*. The invention claims that it can be used as an adjunct therapy for treating fever, bronchitis, eye and ear infections, scurvy, skin infections, and arthritis, as well as for lowering blood pressure and blood sugar. Additionally, it is said to help alleviate fatigue and anxiety and improve sleep [208]. In 2004, Ranjitsinh Solanki patented a composition of 11 herbs intended for cancer treatment, with *M. oleifera* included at a concentration of 8–12%. The patented formula was effective against squamous cell carcinoma and lung cancer cells [209].

In 2005, Suman Preet Singh Khanuja patented a glycoside isolated from the plant’s pods, called niaziridine. The invention enhances the activity of antibiotics, such as rifampicin, tetracycline, and ampicillin. The patent claims that this biomolecule increases the absorption of certain drugs and vitamins through gastrointestinal membranes, thereby improving their bioavailability [210]. In 2011, Richard Gomez patented a dietary supplement containing *M. oleifera*, *Curcuma longa*, and piperine, which has been shown to aid in weight reduction [209]. Anti-adipogenic and lipolytic compositions have also been developed, containing extracts from *Curcuma longa* (10%) and *M. oleifera* (30–70%). It has been demonstrated that the supplement can combat obesity. A patent was published in 2013 [210].

## 12. Interactions Between *M. oleifera* and Pharmaceutical Drugs

### 12.1. Synergistic Effects

Due to resistance to chemotherapy drugs, cancer cell recurrence is often observed. It is known that some plant extracts have antitumor and immunoregulatory effects with relatively low toxicity [4]. They show that the chemotherapy drug doxorubicin, combined with *M. oleifera* leaf extracts, has a synergistic effect in inhibiting the growth of HeLa cells. Compounds with biological activity in the methanol extract from the bark can enhance the action of antibiotics, helping to overcome resistance in multidrug-resistant organisms. Vankwani, S. et al. demonstrated a significant synergistic effect between *M. oleifera* stem bark extract and the antibiotic ampicillin against methicillin-resistant *S. aureus* [209]. Abu-Hussien et al. reported that the combination of essential oil from *M. oleifera* seeds with essential oils from cinnamon (*Cinnamomum verum*) and black cumin (*Nigella sativa*), significantly increased antibacterial activity against *Staphylococcus aureus*, and the inhibitory concentration values for the combination were lower compared to the individual oils [210]. In an animal model, the combination of *M. oleifera* extract with fluoxetine, a selective serotonin reuptake inhibitor, produced enhanced antidepressant-like effects compared to either treatment alone. This suggests a potential synergistic interaction, although further research is needed to confirm these findings in humans [211].

### 12.2. Pharmacokinetic Drug Interactions

- Antimalarial drugs. An experimental study demonstrated that co-administration of *M. oleifera* leaf extract with chloroquine resulted in antagonistic effects, potentially due to the inhibition of chloroquine absorption. This interaction may reduce the drug efficacy in treating malaria, highlighting the need for caution when combining Moringa with certain antimalarial drugs [212].
- Cytochrome P450 enzyme inhibition. In vitro studies have shown that *M. oleifera* extracts can inhibit cytochrome P450 enzymes, particularly CYP3A4 and CYP2D6, which are involved in the metabolism of many drugs. The degree of inhibition varied depending on the extract type and concentration, with methanolic leaf extracts showing more potent effects [213].
- Antihypertensive drugs. Compared to standard antihypertensive drugs, *M. oleifera* leaf extract administered alone to spontaneously hypertensive rats reduced blood pressure. However, when combined with these drugs, no synergistic effects were observed. This suggests that the concurrent use of *Moringa* with antihypertensive medications may not enhance therapeutic outcomes [214].

## 13. Conclusions

*M. oleifera* is a plant of exceptional nutritional and pharmacological significance, known for its rich profile of vitamins, minerals, amino acids, and bioactive secondary metabolites. Extensive in vitro and in vivo studies have demonstrated its antioxidant, anti-inflammatory, antimicrobial, antidiabetic, hepatoprotective, neuroprotective, and anticancer properties, supporting its integration into food, medicinal, and cosmetic formulations. Its utility as a functional food and dietary supplement is further enhanced by its safety profile and wide-ranging biological activities. Moreover, *M. oleifera* is a fast-growing, drought-resistant species that thrives in poor soils, making it an ecologically sustainable crop suitable for cultivation in arid and semi-arid regions. Its resilience and low agricultural input requirements make it especially valuable for improving food security and supporting local economies in resource-limited settings. Future research should focus on standardizing extraction methods, elucidating underlying molecular mechanisms, and conducting well-designed clinical trials to validate efficacy and safety. Integrating *M. oleifera* into public health strategies and evidence-based therapeutics holds great promise for addressing nutritional deficiencies and managing chronic diseases on a global scale.

## Figures and Tables

**Table 1:** Common names of *M. oleifera* in different countries and languages.

**Country**	**Language**	**Common Name(s)**
Arab countries	Arabic	rawag
Bangladesh/India	Bengali	sujina, sohjna, sajina
East Africa	Swahili	mronge, mzunze, mlonge, mrongo
Ethiopia	Amharic	shiferaw
France	French	acacia blanc, neverdie, moringa ailé, ben ailé, pois quenique
Germany	German	pferderettichbaum, meerrettichbaum
Haiti/Martinique	Creole	patois
Hong Kong	Cantonese	nuge
India	Hindi	sanjna, suhujna, sondna, sohanjna, shajna, munga ara, sainjna, mungna
Laos	Lao	‘ii h’um
Latin America	Spanish	paraíso blanco, paraíso francés, reseda
Malaysia	Malay	sajina, merunggai
Myanmar	Burmese	dan-da-lun, dandalonbin
Nepal	Nepali	shobhanjan, sohijan
Nigeria	Yoruba	ewe-igbale
Pakistan	Urdu	sahjnao
Thailand	Thai	makhonkom, ma-rum, phakihum
USA/UK/Other	English	moringa tree, ben-oil tree, cabbage tree, clarifier
Vietnam	Vietnamese	chùm ngây

**Table 2:** Taxonomic classification of *M. oleifera* Lam.

Taxonomic Rank	Classification
Kingdom:	Plantae
Subkingdom:	Tracheobionta
Superdivision:	Spermatophyta
Division:	Magnoliophyta
Class:	Magnoliopsida
Subclass:	Dilleniidae
Order:	Capparales
Family:	Moringaceae
Genus:	*Moringa*
Species:	*Oleifera*

**Table 3:** Vitamin content in *M. oleifera* Lam. leaves.

Vitamins	mg/100 g		
	Raw Leaves	Dried Leaves	Leaf Powder
A	1.28	3.63	16.30
B1	0.06	2.02	2.64
B2	0.05	21.30	20.50
C	220.00	15.80	17.30
E	448.00	10.80	113.00

**Table 4:** Mineral content in *M. oleifera* Lam. Leaves [47].

Minerals	mg/100 g		
	Fresh Leaves	Dried Leaves	Leaf Powder
Calcium	440.00	2185.00	2003.00
Potassium	259.00	1236.00	1324.00
Magnesium	42.00	448.00	368.00
Phosphorus	70.00	252.00	204.00
Iron	0.85	25.60	28.20

**Table 5:** Distribution of major phytochemical constituents in different parts of *M. oleifera*.

Class Compounds	Plant Part	Phytoconstituent
Flavonoids	Leaves	Apigenin, Apigenin-*O*-8-glucoside, Apigenin-7-C-glucoside, Astragalin, Daidzein, Genistein, Isoquercitrin, Isorhamnetin, Isorhamnetin 3-*O*-(6″-malonylglucoside), Kaempferide-3-*O*-(2″-*O*-galloyl rhamnoside), Kaempferol, Kaempferol-3-*O*-β-D-(6″-*O*-malonyl)-glucoside, Luteolin, Myricetin, Quercetin, Quercetin-3-acetylglucoside, Quercetin-*O*-3-glucoside, Quercetin-*O*-3,7-diglucoside, Rutin
	Flowers	Rhamnetin, Isoquercitrin, Kaempferitrin
Carbamates	Leaves	Niazinin A, Niazinin B, Niazimicin, Niazimimin A, Niazimimins B, Marumoside A, Marumoside B, Pterygospermin
	Pods	Niazicin A, Niazidin, Niazinin A, *S*-Methyl-*N*-thiocarbamate, Pterygospermin
	Seeds	*O*-*n*-Butyl-4-[(α-l-rhamnopyranosyloxy)benzyl]thiocarbamate, *O*-Ethyl-4-[(α-L-rhamnopyranosyloxy)-3-hydroxybenzyl]thiocarbamate, *N*-[4-(β-l-Rhamnopyranosyl)benzyl]-1-*O*-α-d-glucopyranosyl-thiocarboxamide
	Roots	1,3-Dibenzyl urea
Phenolics	Leaves	Sinapic acid, Gentistic acid, Syringic acid, Chlorogenic acid, Cryptochlorogenic acid, 4-*O*-caffeoyl quinic acid, 5-*O*-caffeoyl quinic acid, Epicatechin,
	Seeds	Gallic acid, *p*-Coumaric acid, Ferulic acid, Caffeic acid, Protocatechuic acid, Vanillin, Ellagic acid, Catechin, Moringyne
	Stems	4-Hydroxymellein, *p*-Hydroxybenzoic acid, *p*-Hydroxybenzaldehyde, *trans*-Ferulic acid, Lasiodiplodin
	Rootbark	*p*-Hydroxybenzaldehyde, De-*O*-methyllasiodiplodin
Glucosinolates	Leaves	Niazirin, Niazirinin
	Pods	Sulforaphane, Methyl-1-aminopentasulfide-5-sulfinate
	Seeds	Niazirin, Glucomoringin, Glucosinalbin, Glucoraphanin, Glucoiberin, Glucobarbarin
	Roots	4-*O*-(α-l-Acetylrhamnopyranosyloxy)-benzyl glucosinolate

**Table 6:** Antioxidant activity of different parts of *M. oleifera* using various solvent extracts and assays.

Plant Part	Solvent Type	Assay	IC_50_	References
Leaves	Petroleum ether	DPPH	42.56 μg/mL	[64]
	Ethyl acetate	DPPH	5.72 μg/mL	[64]
	Ethanol	DPPH	1.87 mg/mL	[56]
	Ethanol	ABTS	1.36 mg/mL	[56]
	Methanol	DPPH	387.00 µg/mL	[61]
	Crude methanol	DPPH	35.42 μg/L	[64]
Dry leaves	Methanol	FRAP	396.43 μmol TE/g	[65]
Dry leaves	Methanol	ORAC	3197.24 μmol TE/g	[65]
Stems	Methanol	DPPH	1116.00 µg/mL	[61]
Roots	Ethanol	DPPH	3.31 mg/mL	[56]
Roots	Ethanol	ABTS	1.24 mg/mL	[56]
Seeds	Ethanol	ABTS	40.35 mg/mL	[56]
	Crude methanol	DPPH	91.13 μg/mL	[64]

**Table 7:** Summary of in vivo studies on the anti-inflammatory activity of *M. oleifera* extracts.

Extract Type	Animal Model/Dose	Observed Effects	References
Aqueous leaf extract	200 mg/kg (rat paw edema)	Inhibition of inflammation comparable to ibuprofen (40 mg/kg) using egg albumin-induced edema model	[48]
Aqueous leaf extract	424 mg/kg (rat paw edema)	Similar anti-inflammatory effect to ibuprofen; egg albumin-induced model	[81]
95% Ethanolic leaf extract	1000 mg/kg (rat paw edema)	Reduced carrageenan-induced paw edema by 79% after 5 h, comparable to diclofenac	[76]
Methanolic leaf extract	250 and 500 mg/kg (guinea pig model)	Anti-asthmatic effect; bronchodilation, ↓ WBC count and histamine in lungs (ovalbumin-sensitized)	[78]

**Table 10:** Commercial dietary supplements based on *M. oleifera* Lam. and their reported applications.

Product	Manufacturer	Plant Part/Extract	Reported Purpose(s)	Reference
Swanson *M. oleifera*	Swanson Health Products, USA	Leaf extract	Antioxidant, supports immune function	[47]
Yango *M. oleifera*	Yango, Poland	Leaf extract	Anticancer and neuroprotective effects	[47]
Vitama Nature *M. oleifera*	Vitamin Nature, Germany	Leaf extract	Hematoprotective, antioxidant	[193]
Jiva Botanicals *M. oleifera*	Jiva Botanicals, USA	Leaf extract	Supports metabolic function	[47]
Natgrown *M. oleifera* leaf	Natgrown, USA	Leaf extract	Regulates blood sugar, antibacterial activity	[193]
*M. oleifera* bark extract capsules	Herbal Hills, India	Bark extract	Hepatoprotective, anticancer effects	[193]
Nature’s Way *M. oleifera* seed	Nature’s Way, Bulgaria	Seed extract	Antidiabetic effects	[193]
Organic *M. oleifera* root extract	Kuli Kuli, Canada	Root extract	Anti-inflammatory properties, immune stimulant	[193]
*M. oleifera* fruit powder	Grenera Nutrients, India	Fruit extract	Supports cardiovascular health	[194]

**Table 11:** Selected cosmetic products containing *M. oleifera* extracts or oil and their reported applications.

Product Description	Plant Part Used	Manufacturer Country	Reported Function	Reference
Wrinkle serum	Seeds	Italy	Anti-wrinkle, smoothing effect	[198]
Natural moringa oil	Seeds	Bulgaria	Hydrates, reduces wrinkles and scars	[199]
Micellar water	Seeds	France	Makeup removal, hydration, soothing	[200]
Moringa body yogurt	Seeds	United Kingdom	Skin hydration and softness	[201]
Facial cleansing foam	Seeds	Bulgaria	Skin purification, pollution removal	[202]
Oil body lotion	Seeds	India	Moisturizing and nourishing	[203]
Facial cleansing foam	Leaves	USA	Gentle cleansing, hydration support	[204]
Anti-aging facial therapy	Leaves	Italy	Hydration, firming, and wrinkle reduction	[198]

## Data Availability

Datasets from the time of this study are available from the respective authors upon reasonable request.

## Abbreviations

ROS	reactive oxygen species
GSH	reduced glutathione
GSSG	oxidized glutathione
ALT	alanine aminotransferase
AST	aspartate aminotransferase
GGT	gamma-glutamyl transferase
COX	cyclooxygenase
BSS	β-sitosterol
MO	*Moringa oleifera*
LPS	lipopolysaccharide
DNA	Deoxyribonucleic Acid
iNOS	nitric oxide synthase
MOL	*M. oleifera* leaf
*Mo*-CBP	*Moringa oleifera*-Chitin-binding proteins
NDV	Newcastle disease virus
HSV-1	Herpes Simplex Virus Type 1
ALP	alkaline phosphatase
TNF	tumor necrosis factor
p.os	orally
STZ	streptozotocin
LDL	Low-density lipoprotein
HFD	follicle-stimulating hormone
mRNA	Messenger Ribonucleic acid
